# Tauopathy promotes spinal cord-dependent production of toxic amyloid-beta in transgenic monkeys

**DOI:** 10.1038/s41392-023-01601-6

**Published:** 2023-09-22

**Authors:** Zhuchi Tu, Sen Yan, Bofeng Han, Caijuan Li, Weien Liang, Yingqi Lin, Yongyan Ding, Huiyi Wei, Lu Wang, Hao Xu, Jianmeng Ye, Bang Li, Shihua Li, Xiao-Jiang Li

**Affiliations:** 1 Guangdong Key Laboratory of Non-human Primate Research, Key Laboratory of CNS Regeneration (Ministry of Education), GHM Institute of CNS Regeneration, Jinan University, Guangzhou, 510632 China; 2 Center of Cyclotron and PET Radiopharmaceuticals, Department of Nuclear Medicine and PET/CT-MRI Center, the First Affiliated Hospital of Jinan University, Guangzhou, 510630 China; 3 Guangdong Landau Biotechnology Co. Ltd., Guangzhou, 510555 China

**Keywords:** Diseases of the nervous system, Cell death in the nervous system

## Abstract

Tauopathy, characterized by the hyperphosphorylation and accumulation of the microtubule-associated protein tau, and the accumulation of Aβ oligomers, constitute the major pathological hallmarks of Alzheimer’s disease. However, the relationship and causal roles of these two pathological changes in neurodegeneration remain to be defined, even though they occur together or independently in several neurodegenerative diseases associated with cognitive and movement impairment. While it is widely accepted that Aβ accumulation leads to tauopathy in the late stages of the disease, it is still unknown whether tauopathy influences the formation of toxic Aβ oligomers. To address this, we generated transgenic cynomolgus monkey models expressing Tau (P301L) through lentiviral infection of monkey embryos. These monkeys developed age-dependent neurodegeneration and motor dysfunction. Additionally, we performed a stereotaxic injection of adult monkey and mouse brains to express Tau (P301L) via AAV9 infection. Importantly, we found that tauopathy resulting from embryonic transgenic Tau expression or stereotaxic brain injection of AAV-Tau selectively promoted the generation of Aβ oligomers in the monkey spinal cord. These Aβ oligomers were recognized by several antibodies to Aβ1–42 and contributed to neurodegeneration. However, the generation of Aβ oligomers was not observed in other brain regions of Tau transgenic monkeys or in the brains of mice injected with AAV9-Tau (P301L), suggesting that the generation of Aβ oligomers is species- and brain region-dependent. Our findings demonstrate for the first time that tauopathy can trigger Aβ pathology in the primate spinal cord and provide new insight into the pathogenesis and treatment of tauopathy.

## Introduction

Microtubule-associated protein tau, which is encoded by the MAPT gene, is highly expressed in neuronal axons and participates in microtubule assembly and function of axons.^1–3^ Pathologic or genetic alterations in tau lead to its hyperphosphorylation and intracellular accumulation to form neurofibrillary tangles (NFTs) in the brains of a variety of neurodegenerative diseases including Alzheimer’s disease (AD), progressive supranuclear palsy (PSP), corticobasal degeneration (CBD), argyrophilic grain disease (AGD), Pick’s disease (PD), as well as hereditary frontotemporal dementias (FTDs).^4,5^ Thus, human tauopathies are the pathological hallmark of a heterogeneous group of diseases that present diverse neurological phenotypes ranging from movement disorders to dementia. The neuropathological features in AD are characterized by tauopathy and the extracellular plaques composed of β-amyloid (Aβ) aggregates.^6–8^ The development of these two well-established pathological hallmarks is well correlated with the clinical disease severity,^9,10^ and it has been widely accepted that Aβ accumulation leads to tauopathy in the late stage of diseases,^4,10,11^ However, it has also been recognized for long time that depletion of tau is protective against Aβ-mediated deficits.^12–15^ Despite these interesting findings, it remains to be investigated whether tauopathy can act as the primary cause to trigger Aβ-related neuropathology.

Animal models are vitally important for investigating the pathogenesis of tauopathy. The identification of mutations in the *MAPT* gene in FTD^5,16^ has led to great efforts on the generation of transgenic animal models to elucidate the role of Tau in neurodegeneration. Transgenic Tau animal models, especially rodent models, remarkably recapitulate tauopathy and have provided important insight into the pathogenesis of tauopathy-related diseases.^17–21^ However, Tau is expressed through alternative splicing in the human brain, resulting in six isoforms containing three repeats (3 R) or four repeats (4 R), whereas only 4 R Tau is expressed in the adult rodent brain.^22–24^ It is known that tauopathy also occurs in the aged brains of non-human primates but is absent in the brains of old small animals including rodents.^25–28^ Furthermore, human Tau consists of 11 primate-specific amino acids, which can be found in macaques but not in rodents.^29^ There are a large number of phosphorylation sites (at least 40 serine/threonine and two tyrosine sites) in human Tau^30–32^ and considerable disparity in phenotypes of a variety of Tau transgenic animal models.^33^ Considerable differences exist in brain development, anatomical structure, cognitive and behavioral complexity between small and large animals. These differences explain why many human neurological and neuropsychiatric diseases are inadequately modeled in small animals. The primate brain, with its increased neuronal number and larger cerebral cortex, possesses more complex neural architectures that determine cognitive abilities. These differences can also impact the pathological and behavioral phenotypes of animal models used in neurodegenerative disease research. Aging processes also vary significantly among different animal species. Rodents, for example, typically have a lifespan of less than 3 years, which may not be sufficient for the occurrence of neurodegeneration or other important pathological changes that usually take decades to develop in humans. The rapid development and accelerated aging in small animals could make their brains or neuronal cells less vulnerable to insults or toxic proteins, resulting in a lack of robust neurodegeneration despite the presence of impaired brain function.

Therefore, it is logical and important to utilize non-human primates to investigate the role of human Tau and its related pathology, given their closer resemblance to human brain structure and function. In our early studies, we employed a CRISPR/Cas9 targeting approach to generate genetically modified monkeys for the purpose of modeling significant brain diseases.^34–36^ Our findings indicate that monkey models more accurately replicate neuropathology observed in patient brains and offer valuable insights into disease pathogenesis that may not be obtained from small animals. For instance, monkeys with mutations in the PINK1 gene, which can lead to early onset Parkinson’s disease, exhibit neurodegeneration, whereas PINK1 knockout mice do not display such degeneration.^35^ Similarly, monkeys with mutations in the CHD8 gene, which are responsible for autistic behaviors and macrocephaly in humans, exhibit abnormal gliogenesis, a phenomenon absent in mutant mouse models.^36^ These discoveries provide compelling evidence for the use of non-human primates as an essential model for investigating neuropathology and pathogenesis-related to AD.

Given the absence of any reports on the creation of transgenic Tau monkeys through embryonic transgenic expression, it is imperative to establish such a monkey model to investigate tauopathy and its relationship with Aβ accumulation. In the current study, we established non-human primate models that express mutant human *Tau* P301L (0N4R P301L) via embryonic lentiviral transduction and stereotaxic brain injection of AAV. We found that mutant Tau elicited neurodegeneration accompanied by typical features of tauopathy and motor function deficits. Moreover, transgenic Tau selectively promotes the production of toxic Aβ oligomers in the spinal cord of Tau transgenic monkeys and adult monkeys injected with AAV-Tau (P301L). Our studies demonstrated for the first time that Tau can cause the formation of endogenous toxic Aβ in a brain region-dependent manner and provide pathogenic insight for how Tau is involved in AD related pathogenesis.

## Results

### Generation of Tau-P301L transgenic (Tau) monkeys

**Fig. 1:**
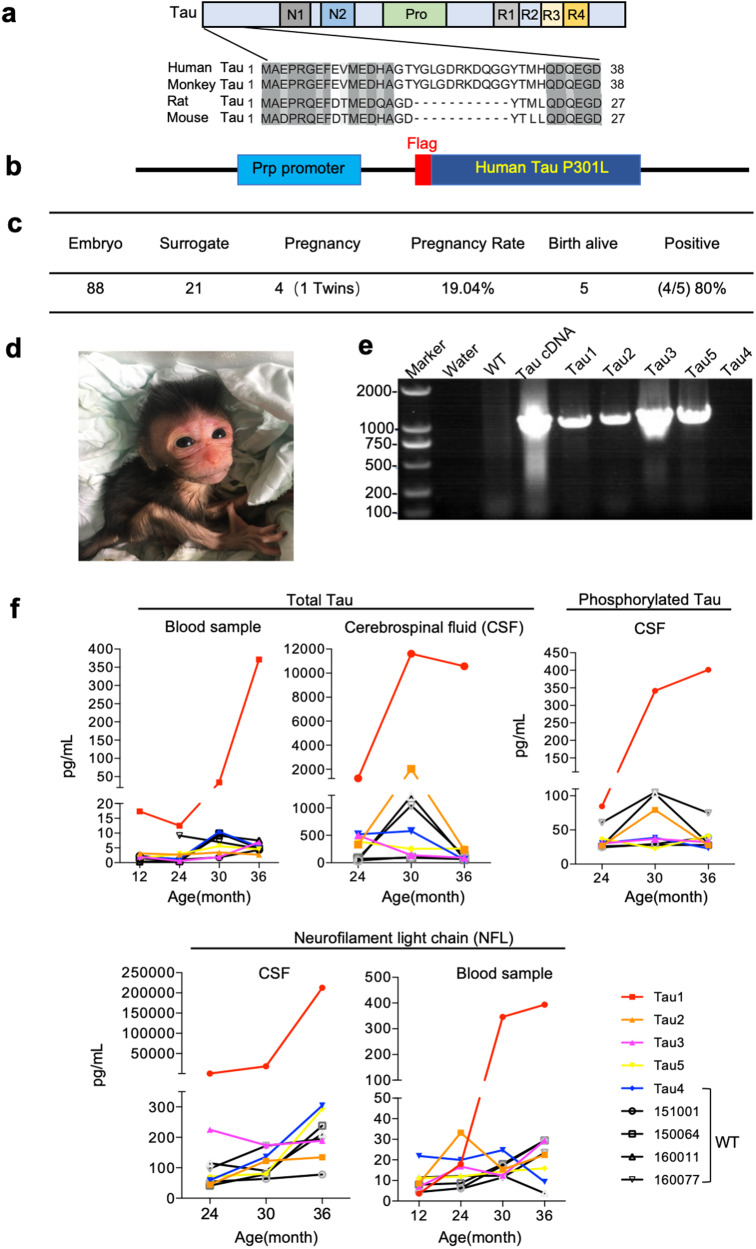
Generation of Tau-P301L transgenic (Tau) monkeys. **a** Schematic diagram of the N-terminal Tau proteins of the primates and rodents. **b** Transgenic Tau (Tau-P301L) is tagged with the Flag epitope at its N terminus and expressed under the control of mouse Prp promoter. **c** Summary of embryo transfer, pregnancies, and newborn monkeys. Injected embryos (88) were transferred to 21 monkey surrogates, resulting in 19.4% pregnancy with 5 live birth monkeys and 4 positive (80%) Tau transgenic monkeys. **d** Representative photos of Tau monkey. The photo was taken 1 week after Tau3 monkey was born. **e** Identification of 4 positive transgenic monkeys by PCR analysis of the blood. WT monkey genome was used as negative control and plasmids were used as positive control. **f** Levels of AD-associated biomarkers including total Tau, p-Tau, and NFL in plasma and cerebrospinal fluid at different ages

Comparison of Tau sequences in different species shows that the primate Tau contains additional 11 amino acids in the N-terminal region when compared with the rodent Tau (Fig. 1a). To establish a monkey model expressing mutant human Tau, we used monkey embryonic injection of transgene as described in our early studies.^34–36^ We injected 88 mature cynomolgus (*Macaca fascicularis*) oocytes with high titer (10^7^ vg ml^−1^) lentiviruses expressing human MAPT gene with *Tau* p301L mutation (0N4R P301L) that was also tagged with the Flag epitope at the N terminus and expressed under the control of the mouse prion promoter (Fig. 1b). These oocytes were then intracytoplsmically injected with sperm for i*n vitro* fertilization. Embryos at 4–8 cell stages were transferred to 21 surrogates, resulting in 4 pregnancies that gave rise to 5 (Tau1-Tau5) newborn monkeys (Fig. 1c, d). PCR analysis of the blood samples revealed that 4 of these newborn monkeys carried the transgenic Tau gene in their blood cells (Fig. 1e), though the expression of transgenic Tau in their brains remains unknown. Genome-wide distribution of transgene analysis showed that Tau1 monkey carried the greatest numbers of insertions or copies of transgenic *Tau* than other monkeys (Supplementary Fig. 1a, b).

### Abnormal MRI and motor dysfunction in Tau monkeys

**Fig. 2:**
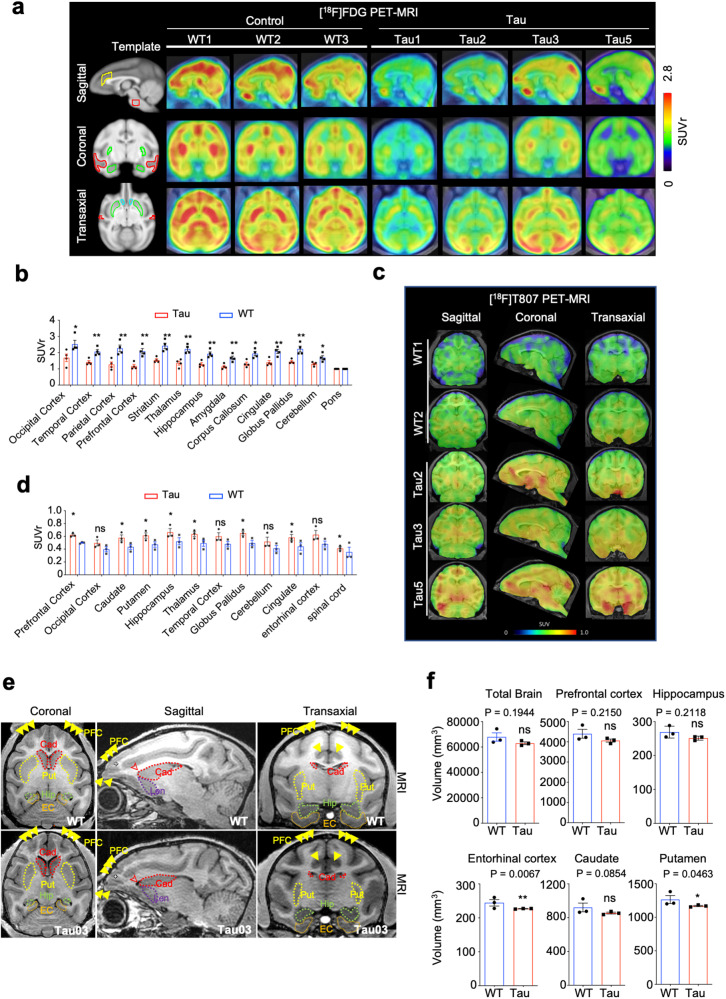
Decreased glucose metabolism and increased Tau protein deposition in the Tau monkey brains. **a**, **b** Evaluation of [^18^F] FDG in WT and Tau monkeys by PET-MRI imaging. Patterns of brain regions (the left panel) and representative brain PET images overlaid on their individual MRI are presented. Regional radioactivity was normalized to the injected radioactivity and body weight and expressed as the SUVr with Pon uptake as a reference. Regional SUVr values in different brain regions in WT (*n* = 3) and Tau monkeys (*n* = 4). Data were obtained from 30-month-old monkeys by PET-MRI imaging tests, presented as mean ± SEM, and were analyzed by Student’s *t* test. NS, *P < 0.05, **P < 0.01. **c**, **d** Tau protein deposition detected by PET-MRI technology with Tau protein tracer of [^18^F]T807 was increased in the brain of Tau monkeys. Regional radioactivity was normalized to the injected radioactivity and body weight and expressed as the SUVr with Pon uptake as a reference. Regional SUVr values in different brain regions in WT (*n* = 3) and Tau monkeys (*n* = 3). Data were obtained from 38-month-old monkeys by PET-MRI imaging tests, presented as mean ± SEM, and are analyzed by Student’s *t* test. NS, *P < 0.05, **P < 0.01. **e**, **f** Representative photos of MRI images (**e**) and quantification of MRI results (**f**). MRI analysis reveals T2-weighted coronal images of live WT (*n* = 3) and Tau (*n* = 3) monkeys at ~36 months old. The total brain volume and sub-region volume of the prefrontal cortex, hippocampus, entorhinal cortex, caudate, and putamen, were presented. Data were analyzed by Student’s *t* test and presented as mean ± SEM. *p < 0.05; **p < 0.01

Transgenic newborn monkeys developed normally and did not show obviously abnormal behaviors before the age of 28 months. We collected their CSF at different ages from 12 to 36 months and found that Tau1 monkey had the highest level of Tau, phosphorylated Tau, and neurofilament light chain (NFL) that was also evidently high in the blood sample (Fig. 1f). However, transgenic Tau monkeys did not display any high levels of aβ40 and aβ42 in their plasma and cerebrospinal fluid when compared with control WT monkeys (Supplementary Fig. 1c). It is possible that Tau-mediated alterations in other biomarkers may be age-dependent and occur in the aged monkeys. We previously established PET-MRI assay that can offer labeling specific molecules at high resolution in the brain.^37^ Using this assay, we performed PET([^18^F] FDG)-MRI analysis of 3 WT and 4 Tau monkeys at the age of 30 months and found that Tau transgenic monkey brains showed reduced [^18^F] FDG uptake to various extents (Fig. 2a), a sign of reduced glucose metabolism indicating decreased neuronal and synaptic activity.^38^ This reduced activity was the most obvious in Tau1, consistent with its highest level of transgenic Tau. Quantification of the relative levels of [^18^F] FDG uptake indicated that the decreased activity was present in various brain regions in Tau monkeys (Fig. 2b). We performed more MRI analysis of monkey brains at the age of 36–38 months based on the availability of PET reagents and the healthy status of transgenic monkeys. Tau1 monkey displayed severe movement difficulty and was therefore not included in the following PET-MRI and behavioral analyses. Using PET-MRI analysis with the radio-labeled probe ([^18^F] T807/AV1451)^39^ for Tau deposition in the monkey brains at the age of 38 months, we found that Tau deposition was more abundant in Tau monkeys than in WT monkeys (Fig. 2c), which was confirmed by quantification of [^18^F]T807/AV1451 signals in different brain regions (Fig. 2d). We also performed additional MRI analysis to compare Tau transgenic and WT monkeys at 36 months of age for brain structure and found that the entorhinal cortex (ERC) and putamen displayed a significant reduction in volume in Tau monkeys (Fig. 2e–f, Supplementary Fig. 2). Although MRI analyses revealed that atrophy is limited in selective brain regions in Tau transgenic monkeys, altered metabolic activity is evident and reflects abnormalities in the early stages of the disease.

**Fig. 3:**
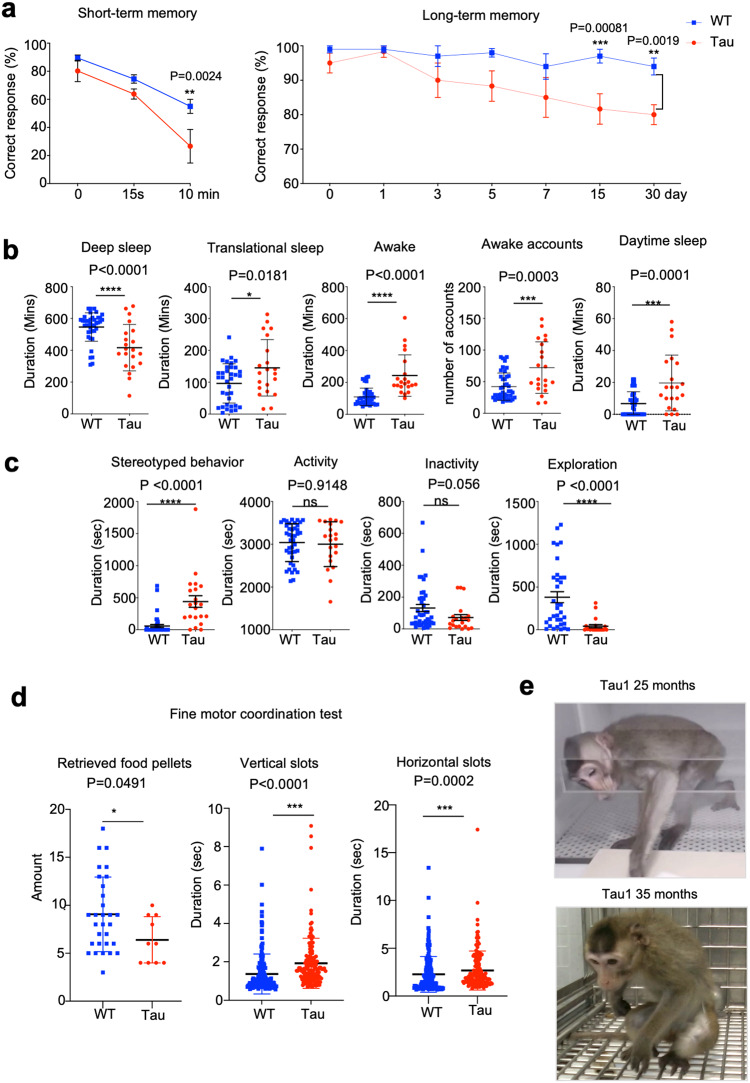
Behavioral phenotypes of Tau monkeys. **a** Impaired cognitive ability of Tau monkeys (*n* = 3) as compared with WT (*n* = 6) monkeys revealed by short-term and long-term memory assays. **b** The sleep pattern of Tau monkeys exhibited abnormalities. The mean duration of deep sleep, translational sleep, and awake periods (measured in minutes per night from 18:00 to 06:00) was compared between Tau and WT monkeys. Tau monkeys spent more time awake than WT monkeys and experienced frequent sleep disruptions (as indicated by a higher number of awake periods per night). Additionally, Tau monkeys had longer periods of sleep during the daytime compared to WT monkeys. The data were collected continuously for 7 days and nights, and the videos were analyzed manually by three experienced technicians. **c** Tau monkeys exhibited stereotypic and anxiety-like behaviors. Tau monkeys showed general activity level, and exhibited less quiet sitting in cages despite no significant difference. Tau monkeys exhibited more stereotypical behaviors and less environment-exploring behaviors than WT controls. Tau (*n* = 3) monkeys and WT (*n* = 6) monkeys were examined. The data were collected from videos between 10 a.m. and 11 a.m. on 7 consecutive days, and the videos were analyzed manually by three experienced technicians. **d** Fine motor coordination test. Hand manual dexterity analysis of the modified Brinkman board task data. Tau monkeys retrieved a fewer number of food pellets from vertical and horizontal slots. Examination of the contact time (CT), defined as the time (duration) of contact between the fingers and the pellet, showed that Tau monkeys had longer CT in both horizontal slots and vertical slots. The behavioral experiments on finger motor coordination test were conducted on multiple trials with three Tau monkeys and six control monkeys. **e** Photos of Tau monkey (Tau1) with no symptoms at 25 months of age and with severe movement difficulty at 35 months of age. Data were analyzed using the repeated measures *t* test. The data are presented as mean ± SEM. *p < 0.05; **p < 0.01; ***p < 0.001

The behaviors of Tau monkeys were compared with those of the age-matched WT monkeys under the same housing conditions. At the age of 38–42 months, we found that Tau monkeys exhibited the delayed response at 10 min in short-term memory test and at 15 and 30 days in long-term memory test (Fig. 3a, Supplementary movie-1 and movie-2). However, more significant alterations were seen in the sleep behavior of Tau monkeys (Fig. 3b, Supplementary movie-3). Also, Tau monkeys exhibited stereotypic and anxiety-like behaviors and less exploration activity (Fig. 3c). Notably, reduced motor function appeared to be significant in Tau monkeys, which was evident in the fine motor coordination test (Fig. 3d, Supplementary movie-4). Tau1 monkey started to show obvious movement difficulty from the age of 28 months, which was progressively worsening till 37 months of age (Fig. 3e, Supplementary movie-5). As a result, Tau1 was euthanized at the age of 37 months for analyzing its brain pathology.

### Neuropathology in Tau transgenic monkeys

**Fig. 4:**
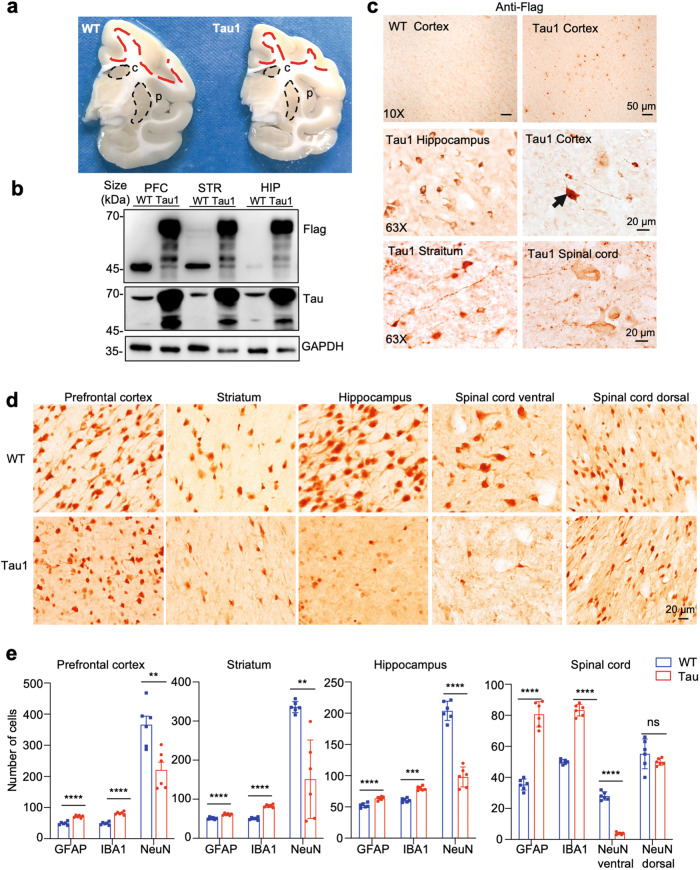
The neuropathology of Tau1 monkey. **a** Gross morphology of the Tau1 monkey (right) brain at 37 months old age showing the globally reduced thickness of the cortex and decreased size of the caudate (c) and putamen (p) as compared with the age-matched WT control (left). **b** Western blot analysis of the Tau protein expression in Tau1 monkey brain tissues. PFC: prefrontal cortex, STR: striatum, HIP: hippocampus. **c** Anti-Flag immunostaining of the prefrontal cortex, striatum, hippocampus, and spinal cord of WT and Tau1 monkey. **d** NeuN staining revealing significant neuronal loss in Tau1 monkey brain region, prefrontal cortex, striatum, hippocampus, and spinal cord. **e** Quantification of the numbers of GFAP, IBA1, and NeuN-positive cells in brain regions (1.34 mm^2^) of WT and Tau1 monkeys (*n* = 6 images/each section from 6 brain sections). Data were analyzed by Student’s *t* test and presented as mean ± SEM. *p < 0.05; **p < 0.01; ***p < 0.001

Gross morphology of the Tau1 brain at the age of 37 months showed the reduced thickness of the cortex and decreased size of the caudate and putamen as compared with an age-matched WT control (Fig. 4a). Western blotting verified the expression of transgenic Tau was expressed in the cortex, striatum, and hippocampus (Fig. 4b). Since transgenic Tau was tagged with Flag, anti-Flag immunocytochemistry was used to unambiguously detect transgenic Tau and revealed specific expression of transgenic Tau in the cortex, hippocampus, striatum as well as spinal cord (Fig. 4c). Hyper-phosphorylated Tau and NFL are important pathological features of tauopathy. Tau1 monkey brain displayed specific staining of phosphorylated Tau (Supplementary Fig. 3a) and NFL (Supplementary Fig. 3b) in various brain regions. Quantification of the numbers of cells that were positively labeled by antibodies to phosphorylated Tau and NFL verified that different brain regions in Tau1 monkey exhibited these two important pathological features (Supplementary Fig. 3c). As expected, transgenic Tau was only detected in the brain tissues but not peripheral tissues because the neuronal prion promoter drives the expression of transgenic Tau (Supplementary Fig. 4a).

NeuN staining of neuronal cells showed a marked reduction of NeuN-positive cells in the ERC of Tau1 monkey (Supplementary Fig. 4b). The reduced density of neuronal cells was also seen in the prefrontal cortex, striatum, hippocampus, spinal cord in Tau1 monkey (Fig. 4d). To assess the effects of transgenic Tau on neuronal and glial cells, we conducted a quantitative analysis of neuronal and glial cell numbers in multiple brain sections from various brain regions in the Tau1 monkey and an age-matched control. The findings demonstrated that the ventral spinal cord exhibited a more pronounced reduction in NeuN-positive cells and an increase in GFAP and Iba1 glial cells compared to other brain regions in the Tau1 monkey (Fig. 4e). Consistent with neurodegeneration in Tau1 monkey, reactive gliosis reflected by increased IBA1 and GFAP staining was seen in the cortex, striatum, hippocampus, and spinal cord in Tau1 monkey as compared with WT monkey (Supplementary Fig. 5a, b).

**Fig. 5:**
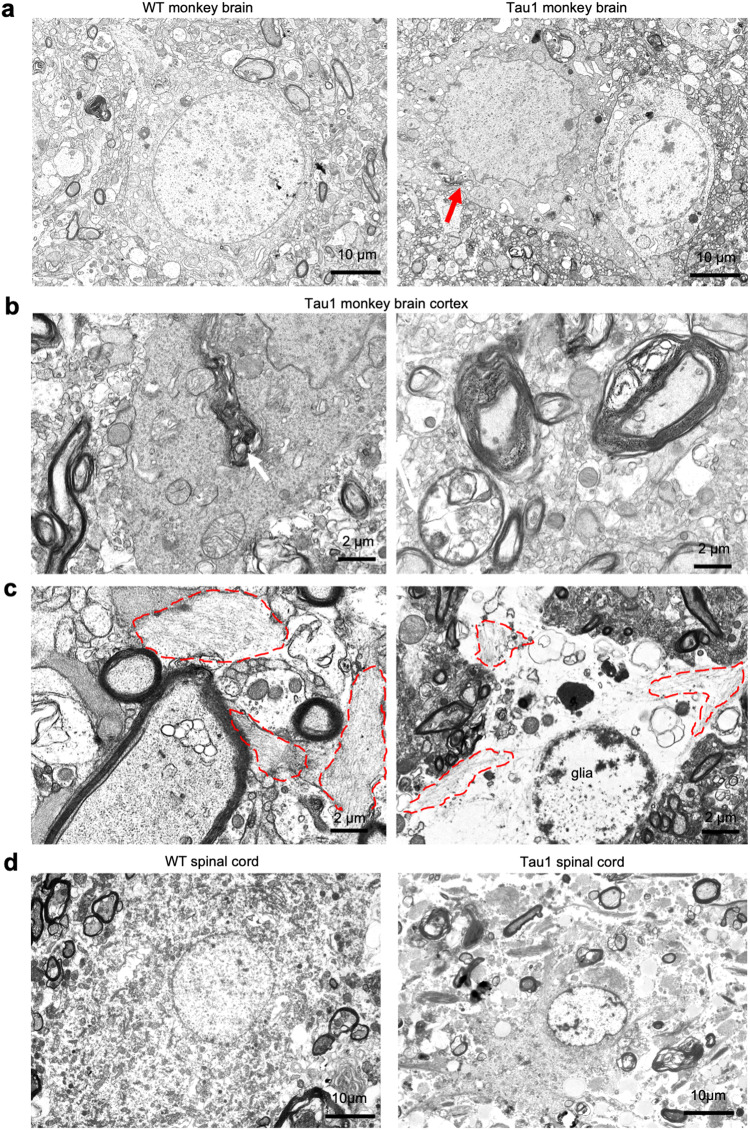
Electron microscopic micrographs of 3-year-old Tau1 monkey brains. **a** The prefrontal cortex of wild-type (WT) monkey contains a neuron showing the clear nuclear membrane and cytoplasmic profiling. The prefrontal cortex in Tau1 monkey showing a dark neuron (red arrow) next to a normal neuron. The dark neuron displays a shrunken appearance, an irregular nucleus without clear ultrastructure, indicating degeneration. **b** In the degenerated dark neuron, degenerated organelles (arrow) without clear profiling are present. In the Tau1 prefrontal cortex, demyelinated axon containing degenerated organelles is evident. Other axons show reduced myelin layers or defective myelin sheath. **c** Neurofibrillary tangles (NFT) enclosed by red dashed lines are visible in a neuron (left) in Tau1 monkey’s hippocampus. In an enlarged microglia-like cell (right), Tau filaments in the areas outlined with red dashed lines are present. The nuclear membrane is not intact and becomes thin. A glial cell is indicated. **d** The spinal cord of wild type (WT) monkey contains a neuron that shows the clear nuclear membrane and cytoplasmic profiling but the spinal cord of Tau1 monkey contains atrophic neurons, indicating degeneration

To obtain ultrastructural evidence for neuropathology, we performed electron microscopic examination of the prefrontal cortex and hippocampus of Tau1 monkey brain. Compared with the age-matched WT control, Tau1 monkey brain displayed dark neurons with the shrunken appearance, irregular nucleus, and lack of clear cytoplasmic organelle ultrastructure (Fig. 5a). In the prefrontal cortex of Tau1 monkey, demyelinated axons with reduced myelin layers or defective myelin sheath and degenerated organelles were evident (Fig. 5b). More importantly, neurofibrillary tangles were visible in neurons and microglial cells (Fig. 5c). In the spinal cord of Tau1 monkey, degenerated neurons (Fig. 5d) and demyelinated axons (Fig. 5e, Supplementary Fig. 6a) were also apparent. All these pathological events indicate that Tau1 monkey had undergone the typic pathological changes similar to those reported in the patient brains with tauopathy.

### Transgenic Tau selectively promoted Aβ oligomer formation in the spinal cord

**Fig. 6:**
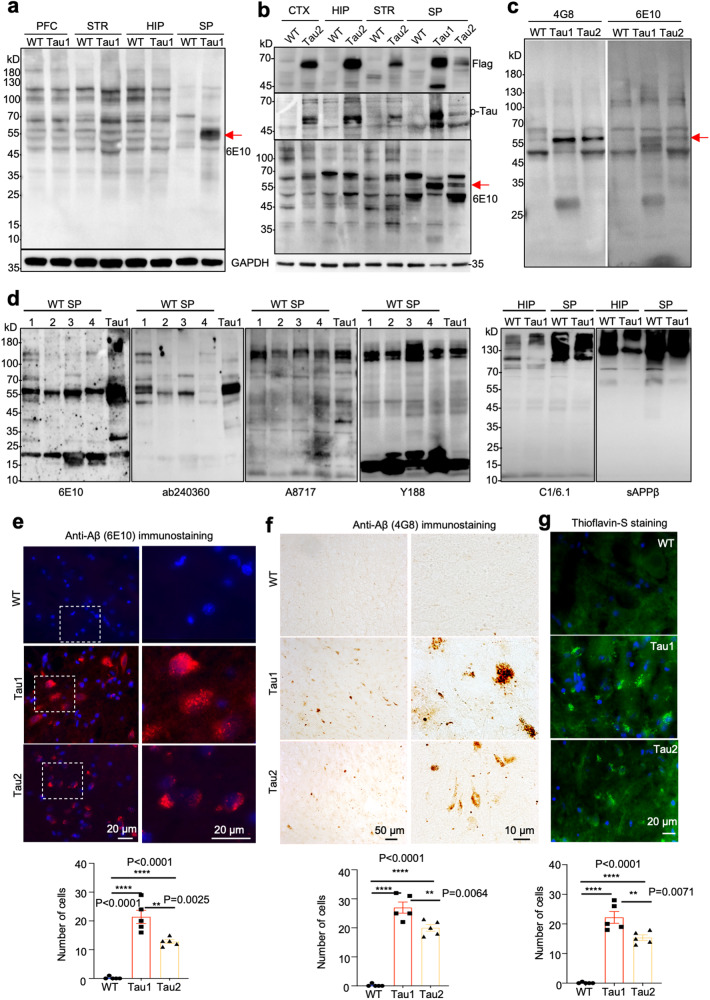
Selective production of toxic Aβ oligomers in the spinal cord of Tau1 monkey. **a** Western blotting of the brain tissues of WT and Tau1 monkey with 6E10 for Aβ42, GAPDH served as a loading control. **b** Western blotting of brain tissues of WT, Tau1, and Tau2 monkeys with antibodies to Flag (upper panel), phosphorylated Tau (middle panel), and 6E10 for Aβ42 (low panel). Red arrow indicates the selective presence of Aβ42 in the spinal cord of transgenic Tau monkeys, whose abundancy corresponds to the level of transgenic Tau. **c** Side-by-side western blotting with two antibodies to Aβ42 (4G8 and 6E10) shows the presence of Aβ42 oligomer in the spinal cord of Tau1 and Tau2 monkeys, though its level is higher in Tau1. **d** Western blotting of monkey brain tissues using different antibodies to Aβ1–42 (6E10 and ab240360), its adjacent region (sAPPβ), and C-terminal APP (A8717, Y188, and C1/6.1). The results show that only anti-Aβ1–42 antibodies recognized Aβ42 oligomer in the spinal cord of Tau1 monkey. **e** Immunofluorescence analysis of the spinal cord of Tau1 and Tau2 monkeys using anti-Aβ42 (6E10). **d** DAB immuostaining of the spinal cord of Tau1 and Tau2 monkeys using anti-Aβ42 (4G8). **f** Thioflavin-S staining of the spinal cord of Tau1 and Tau2 monkeys. **e**–**f** WT monkey served as a control. Quantitation of Aβ42 aggregates is presented beneath each micrograph. Data were analyzed by one-way ANOVA and presented as mean ± SEM. *p < 0.05; **p < 0.01; ***p < 0.001

Because tauopathy and Aβ toxicity often co-exist under various pathological conditions, we examined whether expression of mutant Tau would alter the expression of endogenous Aβ. Interestingly, we found that the spinal cord of Tau1 monkey selectively displayed a high level of a product at MW 54–58 that was reactive to the anti-Aβ1–42 antibody 6E10 (Fig. 6a). Tau2 monkey exhibited much less movement and activity, though not severe as Tau1, and started to lose body weight at 6.5 years of age, leading us to euthanize it to obrain its brain tissues for confirming this important phenomenon. Western blotting also revealed the selective expression of Aβ oligomer in the spinal cord of Tau2, though its level is lower than that in the Tau1 spinal cord (Fig. 6b). When using two different anti-Aβ1–42 antibodies (4G8 and 6E10), we observed the same band of Aβ42 in the spinal cord of Tau1 and Tau2 despite its lower level in Tau2 (Fig. 6c). Consistently, we observed a decreased expression of NeuN in the spinal cord of both Tau1 and Tau2 monkeys (Supplementary Fig. 6b). To verify that the band seen in the spinal cord is Aβ oligomer formed by Aβ1–42, we used multiple antibodies against different regions of APP and Aβ1–42 (Supplementary Fig. 7a, b). Only antibodies to Aβ1–42 (6E10 and ab240360) but not those to the adjacent region (sAPPβ) and C-terminal APP (A8717, Y188, and C1/6.1) were able to detect this Aβ42 product (Fig. 6d), which was named Aβ oligomer in our study.

We then performed immunostaining of the monkey spinal cord and found increased Aβ42 staining by 6E10 in Tau1 and Tau2 monkeys compared with WT monkey (Fig. 6e). Immunostaining with another anti-Aβ42 (4G8) also revealed Aβ aggregates in the spinal corde of Tau1 and Tau2 monkeys (Fig. 6f). Thioflavin-S staining has been used to detect Aβ aggregates,^40^ which also showed increased aggregates in the spinal cord of Tau1 and Tau2 monkeys (Fig. 6g). Thus, using multiple antibodies and different assays (Western blotting, immunostaining, and thioflavin-S staining), we demonstrated the selective presence of Aβ42 immunoreactive products in the spinal cord of transgenic Tau monkeys.

**Fig. 7:**
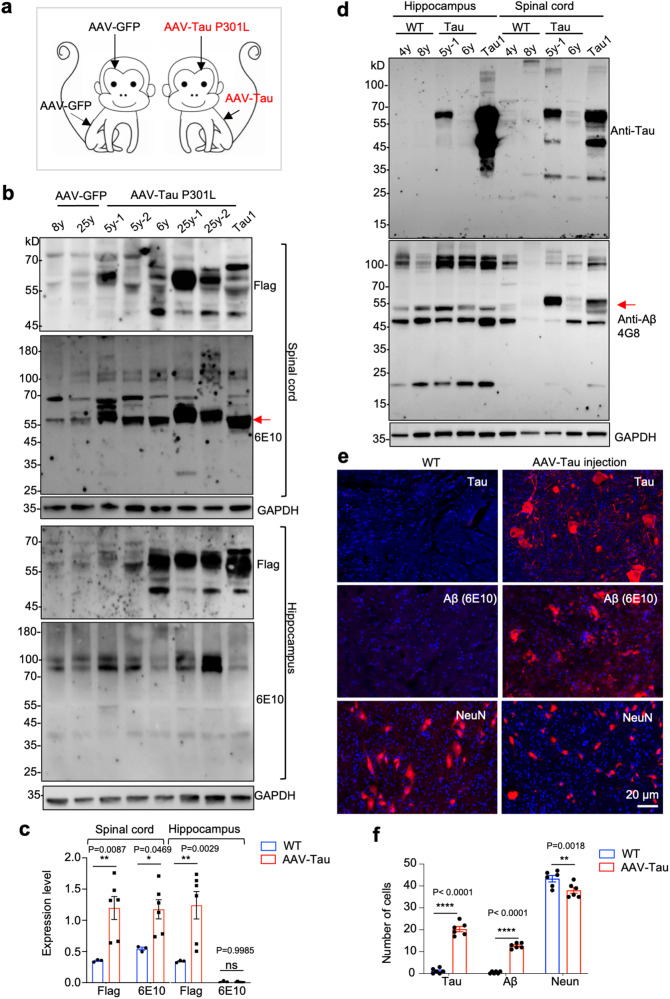
Selective production of toxic Aβ oligomers in the spinal cord of AAV-Tau-injected monkeys. **a** WT monkeys were injected into the brain hippocampus and spinal cord with AAV9-GFP (control) or AAV9-Tau (P301L). **b** Western blotting analysis of the spinal cord (upper panles) and hippocampus (low panels) of AAV-Tau- or AAV-GFP-injected monkeys using anti-flag and anti-Aβ (6E10) antibodies. The spinal cord of Tau1 monkey served as a positive control. 6E10 western blotting shows that the Aβ oligomers (red arrow) were selectively increased in the spinal cord of AAV-Tau-injected monkeys. **c** Quantitation of the relative levels (ratio to GAPDH) of Flag-Tau and Aβ oligomers in Fig. 7b. **d** 4G8 western blotting showing the selective presence of Aβ42 oligomers (red arrow) and its correlation with Tau expression in the spinal cord of AAV-Tau-injected monkeys. **d** Immunofluorescence analysis of the spinal cord of AAV-Tau-injected monkeys using anti-Tau, anti-Aβ (6E10), and anti-NeuN antibodies. WT monkey served as a control. **e** Quantitation of the numbers of Tau, Aβ aggregates, and intact NeuN-positive cells in Fig. 7b. The data are presented as mean ± SEM per image (0.22 mm^2^). Data were analyzed by Student *t* test. *p < 0.05; **p < 0.01; ***p < 0.001

If transgenic Tau induces Aβ oligomer selectively in the spinal cord of monkey, exogenous expression of Tau via AAV should be able to replicate this important phenomenon. Thus, we performed injection of AAV9 expressing mutant Tau (P301L) into the hippocampus and spinal cord of seven WT monkeys at young (5-6 years old) and old (25 years old) ages (Fig. 7a, b). For the control, AAV-GFP was also injected into WT monkeys. Anti-Flag western blotting confirmed the expression of transgenic Tau in the injected spinal cord and hippocampus. However, only the AAV-Tau injected spinal cord showed the production of Aβ oligomer (Fig. 7b). Using WT monkey as control and anti-Tau antibody (6E10), we confirmed that Aβ oligomer was generated in association with transgenic Tau expression in the spinal cord (Fig. 7b). Western blotting with another anti-Aβ42 (4G8) also confirmed the selective expression of Aβ oligomer and its correlation with Tau expression in the spinal cord of AAV-Tau-injected monkey (Fig. 7c). Immunohistochemical staining further revealed that expression of transgenic Tau-induced strong Aβ staining and decreased NeuN staining in the spinal cord (Fig. 7d, Supplementary Fig. 8). If Aβ oligomer was formed by Aβ1–42, the presence of monomer, dimers and other oligomers should also be visible. We, therefore, used more tissue samples and longer exposed western blots with multiple anti-Aβ42 antibodies (D9A3A, ab240360, 6E10, A11). The results indeed showed weak bands of smaller bands that were specifically present in AAV-Tau-injected spinal cord tissues (arrows in Supplementary Fig. 9a), suggesting that other Aβ oligomers might not be as stable as Aβ oligomer at MW between 54–58 kD. Using antibodies to non-Abet42 region in APP (CTF-C1/6.1, sAPPβ), we did not observe specific Aβ42 products in the spinal cord that overexpressed Tau via AAV injection (Supplementary Fig. 9b). We also tested whether AAV-Tau injection could induce Aβ oligomer in the spinal cord in mice but were unable to detect the same Aβ oligomer in mice (Supplementary Fig. 9c). Collectively, these findings suggest that Tau-induced Aβ oligomer formation in the spinal cord is species dependent.

## Discussion

Tauopathy has been found in different transgenic Tau animal models, including a recently reported rhesus monkey model that was injected with AAV-Tau into the vulnerable ERC^41^. However, our monkey models also showed motor impairment, which was seen in some transgenic Tau mouse models.^17,20^ In addition, there are several interesting findings that have not been reported but have important implications for pathogenesis and treatment and therefore are warranted for further discussion.

First, the severe phenotypes observed in the Tau1 monkey are likely associated with its high expression of transgenic Tau, which is supported by the highest level of transgenic Tau found in its cerebrospinal fluid (CSF) and the copy numbers of transgenic Tau. The early death of the Tau1 monkey is also consistent with its severe phenotypes. Similarly, the Tau2 monkey expressed a lower level of Tau in its spinal cord compared to Tau1 and had a longer lifespan with milder phenotypes. It is possible that other Tau transgenic monkeys, which express lower levels of transgenic Tau in their brains, may develop phenotypes when the transgenic Tau accumulates at high levels in their aged brains after several years.

Second, motor dysfunction phenotypes are more obvious and severe than cognitive deficit in Tau transgenic monkeys. The motor phenotype is in line with motor dysfunction in progressive supranuclear palsy (PSP), corticobasal degeneration (CBD), argyrophilic grain disease (AGD), Pick’s disease (PD) in which tauopathy is the major pathological feature.^4,5^ Thus, although abnormally accumulated or overexpressed Tau can participate in many aspects of cellular dysfunction to cause diverse phenotypes, its high level is more likely to severely affect neuronal function related to body movement and motor activity. In support of this possibility, neuronal loss, Tau hyperphosphorylation, and tangle formation were concurrently found in the striatum and spinal cord of Tau1 monkey, which are the brain regions that critically control movement coordination and balance.

Third, the more interesting and important finding is that Tau overexpression can selectively induce Aβ oligomers in the spinal cord in monkeys, which is supported by the following evidence. One is the specific expression Aβ in the spinal cord in transgenic Tau monkeys when compared with other brain regions. Consistently, this specific accumulation is unlikely to cause increases in Aβ levels in the plasma and cerebrospinal fluid in Tau transgenic monkeys. Another is the selective Aβ accumulation in the AAV-Tau-injected spinal cord as opposed to the injected hippocampus. Thus, the fact that both embryonic transgenic and brain injection-mediated expression of transgenic mutant Tau consistently resulted in the selective expression of Aβ demonstrates a spinal cord-dependent formation of Aβ oligomer mediated by tauopathy. This finding is also supported by the lack of altered Aβ42/40 levels in the rhesus monkeys that were injected with AAV-Tau (P301L/S320F) into ERC, a brain region that projects to the hippocampus.^41^ A critical issue is whether Aβ product we observed is an APP fragment or Aβ oligomer that is formed by Aβ1–42. To address this issue, we used multiple antibodies against different epitopes of APP and proved that it is indeed an Aβ oligomer, because it could be recognized by at least 4 antibodies for Aβ1–42 but not by those against the adjacent region or C-terminal APP.

Thus, an immediate and important issue is why only Aβ oligomer, but not monomer, dimers, and other forms of oligomers, was readily detected. We found that overexpression of mutant Tau can induce the brain region (spinal cord) dependent formation of endogenous Aβ oligomer in monkey, which appears to be more stable than other smaller or larger oligomers. It is likely that this particular Aβ oligomer has unique structure or conformation that enables its stability and detection by Aβ 1–42 antibodies via western blotting analysis.

The next question is why Aβ oligomer is only induced in the spinal cord. It is possible that the spinal cord has specific microenvironment that favors the production of small Aβ1–42 while Tau accumulation induces intracellular stress that can promote the oligomerization of Aβ1–42. Another possibility is that Tau overexpression may alter metabolic or gene expression profiling to particularly lead to the generation of Aβ1–42 and its oligomer formation. Indeed, FTD P301L tau transgenic mice show oligomeric and fibrillar species of beta-amyloid (Aβ1–42) that decreased mitochondrial membrane potential in cortical brain cells.^42^ In our studies, we did not observe the production of Aβ oligomer in the cortex of transgenic Tau1 monkey. Also, because AAV-Tau-injected spinal cord in mice did not yield the same Aβ oligomer formation as seen in the monkey spinal cord, we should consider species-dependent differences in Tau-mediated production of Aβ species, which could be related to different posttranslational modifications, interacting partners, and processing of Tau.

Our findings show that pathological Tau accumulation can trigger Aβ oligomer formation, demonstrating for the first time that Tau can play a causal role in endogenous Aβ formation and toxicity. Although neurodegeneration in various brain regions in Tau1 monkey indicates that mutant Tau can mediate neurodegeneration without Aβ oligomer formation, the presence of Aβ oligomer in the spinal cord may generate synergistic toxicity to severely affect motor function, which may explain why Tau1 monkey displayed severe body movement difficulty. Furthermore, the toxicity of Aβ oligomer and its association with tauopathy support the importance in preventing Aβ accumulation for AD treatment and echo the recent successful clinic trial of anti-Aβ drug lecanemab, though whether targeting Aβ can effectively treat AD still remains hotly debated.^43,44^ Since targeting Aβ monomers and plaques was unsuccessful in clinical trials,^45^ it appears that targeting specific forms of Aβ may be important for reducing pathology. Analyses of a large number of human brains across the lifespan have shown that tau pathology begins about a decade before the formation of Aβ plaques.^46,47^ The finding that Aβ deposition was only observed in the spinal cord of the monkey brain in the early disease stage suggests that the spinal cord might be the first site for Aβ protein accumulation. Our findings suggest that Aβ oligomer, which can be induced by mutant Tau, is stable in the spinal cord of non-human primate so that targeting this form of Aβ would be likely beneficial for treating Tau- and Aβ-induced pathology, at least in the spinal cord.

## Materials and methods

### Animals

Adult healthy female cynomolgus monkeys were housed in individual cages at Yuanxi Biotech Inc. Guangzhou and used to generate Tau transgenic monkey in this study. All animal procedures were approved by the Institutional Animal Care and Use Committee (IACUC) at Yuanxi Biotech Inc. Guangzhou. Tau transgenic monkey and age- and gender-matched healthy control cynomolgus monkeys (*M. fascicularis*) were housed in cages and examined for their behaviors at Guangdong Landau Biotechnology Co. Ltd., which is an Association for Assessment and Accreditation of Laboratory Animal Care-accredited facility. All animal-related protocols were approved in advance by the Institutional Animal Care and Use Committee (IACUC) of Guangdong Landau Biotechnology Co. Ltd and Jinan University. This study occurred in strict compliance with the “Guide for the Care and Use of Laboratory Animals (2011)” to ensure the safety of personnel and animal welfare. Health and behavior of the monkeys were monitored daily by the husbandry staff and veterinarians. Animal information is presented in Supplementary information.

### Construction and preparation of lentiviruses carrying the human Tau P301L

The human MAPT cDNA with Tau P301L mutation (0N4R P301L) encoding 373 amino acids was tagged with flag at the N terminus and was inserted into a lentiviral vector for monkey-fertilized egg injection. The lentiviral vector contains the mouse prion promoter,^48^ which drives the expression of transgenic Tau. The same human MAPT cDNA was also expressed via adeno-associated virus vector AAV9 for stereotaxic brain injection. Virus vectors were produced by standard protocols and provided at a titer of 10^7^ vg /ml for lentiviruses and 10^13^ vg /ml for AAV-Tau by PackGene Biotech. Co. Ltd.

### Production of cynomolgus monkey oocytes, embryos, and babes

Assisted reproductive techniques in cynomolgus monkeys have been described in our previous study.^34,35^ In brief, adult females were hormone stimulated, and their oocytes were recovered for in vitro fertilization and culture. Metaphase-II arrested oocytes were selected for perivitelline space injection of lentiviral solution. After virus injection, the oocytes were fertilized by intracytoplasmic sperm injection, followed by in vitro culture. Embryos at the 4–8 cell stage were selected for embryo transfer based on morphological appearance. Surrogate females at synchronized reproductive cycles were identified based on their menstrual cycle. Pregnancy was confirmed by B-ultrasound 30–35 post-transplantation days. The surrogates delivered newborn monkeys naturally after around 160 days of gestation.

### CSF and plasma sample collection and biomarker measurements

The CSF sample collection and assay followed standard protocols and have been described previously.^49^ In short, after monkeys fasted for at least 8 h, a lumbar puncture was performed at the intervertebral space L3-L4 or L4-L5 using a standard needle. CSF was collected into a 1.5 ml sterile microtube (Axygen, MCT-150-C) and immediately frozen at −80 °C. Blood samples were obtained on the same day as the lumbar puncture in fasting conditions. Whole blood was drawn with a 20 G needle gauge into a 5 ml EDTA tube. Tubes were gently inverted 5–10 times and centrifuged at ×3000 *g* for 10 min at 4 °C. The supernatant was aliquoted in volumes of 200 μl into EP tubes and immediately frozen at −80 °C. The samples were processed at room temperature. The time between collection and freezing of both CSF and plasma sample was <30 min. All CSF and plasma biomarkers including total tau, p-tau231, NFL, Aβ40, and Aβ42 were analyzed by Simoa platform of G-Bio Co., LTD, Hangzhou, China.

### Western blot analysis

Primary antibodies to Flag (Sigma, F1804), NeuN (Abcam, ab177487) GFAP (Invitrogen,13–0300), IBA1(WAKO,019–19741), β-Amyloid,1–16 (6E10, Biolegend, SIG-39320), β-Amyloid, 17–24 (4G8, Biolegend, SIG-39200), β-Amyloid 1–42(mOC98, Abcam, ab240360), Amyloid Precursor (Y188, Abcam, ab32136), Amyloid Precursor (Sigma, A8717), APP C-Terminal fragment (C1/6.1, Biolegend, SIG-39152), GAPDH (Proteintech, 60004-1-Ig), Neurofibrillary tangles (Abcam, ab136407), Tau (Tau5, Abcam,ab80579), Phospho-Tau (AT8, Invitrogen, MN1020), β- Amyloid 1–42 (Cell Signaling Technology, 14974 S), sAPPβ(Biolegend, SIG-39138), and Oligomer (A11, Invitrogen, AHB0052) were used. For western blotting, Tau1 and WT tissues were lysed in ice-cold RIPA buffer (50 mM Tris, pH 8.0, 150 mM NaCl, 1 mM EDTA pH 8.0, 1 mM EGTA pH 8.0, 0.1% SDS, 0.5% DOC, and 1% Triton X-100) containing Halt Protease Inhibitor cocktail (Thermo Scientific) and PMSF. The tissue lysates were incubated on ice for 30 min, centrifuged at 12,000 × *g* for 10 min. The supernatants were loaded to SDS-PAGE, transferred to a PVDF membrane, and blocked with 5% skim milk/PBS for 1 h at room temperature. Primary antibodies were diluted in 3% BSA/TBST (50 mM Tris HCl, pH 7.4 with 20 mM Tween 20) and incubated with the blot membrane overnight at 4 °C. The blotted membrane was washed in TBST 3 times (each time 5 min), followed by incubation with HRP-conjugated secondary antibodies in 5% skim milk/TBST for 1 h at room temperature. After three washes in TBST, ECL plus (ChemiSignal® CLINX, China) was used to detect immunoreactive signals on the membrane.

### Electron microscopy

The procedure of EM examination has been described in our previous studies.^50^ In brief, freshly isolated monkey brain tissue blocks were fixed with 4% paraformaldehyde and 0.2% glutaraldehyde for 48 h and sectioned using a vibratome. All sections used for electron microscopy were dehydrated in ascending concentrations of ethanol and propylene oxide/Eponate 12 (1:1) and embedded in Eponate 12 (Ted Pella, Redding, CA). Ultrathin sections (60 nm) were cut using a Leica Ultracut S ultramicrotome. Thin sections were counterstained with 5% aqueous uranyl acetate for 5 min followed by Reynolds lead citrate for 5 min and examined using a Hitachi (Tokyo, Japan) H-7500 electron microscope.

### Statistical analysis

Statistical significance was assessed using the 2-tailed Student’s *t* test for comparing two groups when the data follows a normal distribution and the variances of the two groups are assumed to be equal. When comparing groups with small sample sizes or when the data are skewed or have outliers, the Mann-Whitney test was employed. When the same subjects were measured under different conditions or at different time points, we used repeated measures t-test. For three or more groups, one-way ANOVA with Tukey’s multiple comparisons tests was used. Calculations were performed with GraphPad Prism software. The quantitative results are presented as mean ± SEM. *P* < 0.05 is considered a significant difference.

### Supplementary information

Supplemental materials

Short-term memory test of WT and Tau3 monkeys at 38 months of age

Long-term memory test of WT and Tau5 monkeys at 40 months of age

Sleep monitoring of WT and Tau2 monkey at 38 months of age

Fine finger coordination assay of WT and Tau3 monkey at 30 months of age

The body movements of Tau1 monkeys at 25 and 35 months of age

## Data Availability

All data associated with this study are present in this paper. Raw data used to generate images and graphs are available from the corresponding author, Z.C.T, upon request.
