# Supplementary material for: Tauopathy promotes spinal cord-dependent production of toxic amyloid-beta in transgenic monkeys
Source: Signal Transduct Target Ther. 2023 Sep 22;8:358. doi: 10.1038/s41392-023-01601-6 (PMC10514290; doi:10.1038/s41392-023-01601-6)
Supplement: Supplementary file 1 — Supplemental materials [file 41392_2023_1601_MOESM1_ESM.docx]

Supplementary Materials for

Tauopathy promotes spinal cord-dependent production of toxic amyloid-beta in transgenic monkeys

Zhuchi Tu1*#, Sen Yan1*#, Bofeng Han1, Caijuan Li1, Weien Liang1, Yingqi Lin1, Yongyan Ding1, Huiyi Wei2, Lu Wang2, Hao Xu2, Jianmeng Ye3, Bang Li1, Shihua Li1, Xiao-Jiang Li1#.

Correspondence to: Zhuchi Tu, email: [atuwater@163.com](mailto:atuwater@163.com); Sen Yang， email： [231yansen@163.com](mailto:231yansen@163.com)； Xiao-Jiang Li， email： [xjli33@jnu.edu.cn](mailto:xjli33@jnu.edu.cn)

**This PDF file includes:**

Materials and Methods

Figures. S1 to S9

Captions for Movies S1 to S5

**Other Supplementary Materials for this manuscript include the following:**

Movies S1 to S5

Materials and Methods

Animal information

| **ID** | **Sex** | **Genotype** | **The ages at which animals were used** | **Enrolled Experiments** |
| --- | --- | --- | --- | --- |
| Tau1 | M | Tau transgenic | Euthanasia at 37 months of age | PET/MRI, Pathology analysis |
| Tau2 | F | Tau transgenic | Euthanasia at 6.5 years of age | PET/MRI, Pathology and behavior analysis |
| Tau3 | M | Tau transgenic | 30-45 months (alive) | PET/MRI, behavior analysis |
| Tau5 | F | Tau transgenic | 30-45 months (alive) | PET/MRI, behavior analysis |
| Tau4 | F | Wild type | 30-45 months (alive) | PET/MRI, behavior analysis |
| 151001 | M | Wild type | 30-45 months (alive) | PET/MRI, behavior analysis |
| 160077 | M | Wild type | 33-45 months (alive) | behavior analysis |
| 160011 | M | Wild type | 33-45 months (alive) | behavior analysis |
| 150064 | F | Wild type | 30-45 months (alive) | PET/MRI, behavior analysis |
| 150084 | F | Wild type | 33-45 months (alive) | behavior analysis |
| 96830 | F | AAV-Tau injection | Retired and euthanasia at 25 year of age | pathology analysis |
| 160129 | M | AAV-Tau injection | Body injury and euthanasia at 5 year of age | pathology analysis |
| 160403 | M | AAV-Tau injection | Body injury and euthanasia at 5year of age | pathology analysis |
| 96690 | F | AAV-Tau injection | Retired and euthanasia at 25 year of age | pathology analysis |
| 150181 | M | AAV-Tau injection | Body injury and euthanasia at 6 year of age | pathology analysis |
| 150057 | M | Wild type | Body injury and euthanasia at 8 year of age | pathology analysis |
| 960510 | F | Wild type | Retired and euthanasia at 25 year of age | pathology analysis |

PCR and genotyping

The blood sample of newborn monkeys were collected via capillary fingertip blood sampling. The genomic DNAs of newborn monkeys were isolated from blood samples using E.Z.N.A. tissue DNA Kit (Omega Bio-Tek, Inc). The primers were designed to amplify DNAs containing the human MAPT gene and adjacent fragment of vector using forward primer, 5'-ATggAAgATCACgCTgggACgT-3', and reverse primer 5'-ATTACTTgTCATCgTCgTCCTTgTAgTCTC-3'. The PCR conditions were 94℃ for 5 min; 94℃ for 30 s, 62℃ for 30 s, 72℃ for 45 s, for 35 cycles; 72℃ for 5 min, and held at 12 ℃.

Immunohistochemistry

Isolated monkey brain tissues were fixed for 24 h in 4% paraformaldehyde/ 0.01 M PBS and then transferred into 30% sucrose to dehydrate at 4℃. The monkey brain tissues were sectioned at 20 mm using a freezing microtome. The slides were fixed for 10 min in 4% paraformaldehyde in 0.01 M PB, pre-blocked in 4% normal goat serum in 0.1% Triton X-100/ PBS for 30 min. Slides were incubated with primary antibodies in 3% BSA/2%NGS/1×TBST overnight at 4℃. Secondary antibodies were added after three washes with PBS. Fluorescence imaging was recorded after covering the slides with mounting buffer. The DAB staining was performed using the Avidin-Biotin Complex kit (Vector ABC Elite, Burlingame, CA, USA). Microscopic images were obtained using a Zeiss LSM 800 confocal microscope and Zeiss Axio Imager A2 inverted scope.

Behavioral analysis

Direct video recording of monkey behaviors and sleep pattern when they were housed individually was performed following our previously protocols^37^. In brief, Three Tau transgenic monkeys and 6 age- and gender- matched control monkeys were observed in cages (L 60 × W70 × H80 cm) with video recording for 7 consecutive days. The behavioral categories of activity, inactivity, environment exploration and stereotypical behaviors were statistically analyzed. The specific definitions of these behaviors can be found in our previous study^37^. The methods analyzing the sleep pattern in cynomolgus monkey have been described elsewhere^51^. Three Tau transgenic monkeys and six age- and gender- matched control monkeys were monitored in cages (L 60 × W70 × H80 cm) with video recording for 7 consecutive nights from 19:00 p.m to 7:00 a.m. In addition to measuring the duration of awake, transitional sleep, deep sleep and waking bouts, we also recorded the duration of daytime sleep in 7 consecutive days. In brief with minor modification, three behavioral states, awake, transitional and deep sleep were scored in 1 min epochs^52^. States lasting < 30 s were not considered, whereas states lasting between 30 and 59 s were rounded to 1 min. The animals were considered to be awake when locomotion occurred, or when over 3 times of body or limb movement occurred within 1 min while in sitting or lying position. Sleep was scored as transitional when 1 or 2 times of body or limb movement occurred within 1 min while in sitting position. During relaxed sleep or deep sleep, the animals always kept the head below the shoulders or bent backward with no body or limb movements when sitting. Deep sleep was also scored when the animal was lying immobile. Sleep duration consisted of all 1 min epochs scored as transitional or relaxed sleep.

Short- and long- term memory test

The delayed response tasks (DRT) are simple, well- established tests that are easy to administer and suitable for assessing spatial, working and episodic memory in NHPs^53,54^. The short-term memory test (STMT) and the long-term memory test (LTMT) were commonly used methods for measuring memory in NHPs, which are often performed using the Wisconsin General Test Apparatus (WGTA). Food reinforcement and food restriction (reduce 15-20%) are commonly used as a means of increasing motivation and positive behavioral output^55^. We conducted two types of DRT in cynomolgus monkey as described previously^56^. Briefly, the subjects were presented with an opaque acrylic sheet with inlaid wells, which were covered with identical opaque acrylic cups or thin plates. Three-wells sheets covered identical acrylic cups were used for STMT while four-wells sheets covered by acrylic plates with different color and shapes were used for LTMT. Food items (‘baits’) were hidden within the wells, and the subjects were allowed to retrieve the baits after a certain time delay. Before the formal experiment was conducted, subjects were trained to master the use of the experimental facility. For STMT, a single bait was hidden in one of the wells and time delays of 0 (no delay), 15 sec, 10 min, were applied. Twelve trials were conducted each time to screen these subjects. For LTMT, the four-well sheets with covers of different colors and shapes were used, and subjects were trained to learn that the baits were only hidden under the well cover with one specific color and shape (e.g., orange cube) before the formal experiment was conducted. A single bait, and longer delays (0, 1, 3, 5, 7, 15, 30 days) were utilized. Six trials were conducted each time to screen these subjects.

Fine finger coordination

The assessment of handedness was based on a palette of behavioral manual dexterity tasks, in which Tau monkeys (n= 3) and control monkeys (n = 6) were enrolled. For monkeys, typical video sequences illustrating the behavioral task described below can be visualized on the modiﬁed Brinkman board task, which has been used routinely for behavioral and motor control studies in macaques^57,58^. The modified Brinkman board for monkeys was made of a rectangular board of Perspex with 50 rounded rectangular slots: 25 slots were oriented horizontally and 25 vertically. Each slot measured 6 mm deep, 14 mm long, and 7 mm wide. The board itself measured 22 cm length, 12 cm wide, and 1.2 cm thick. At the beginning of the test, each slot was ﬁlled with a sugar ﬂavored pellet (diameter 4 mm). The size of slots permitted the monkeys to grasp the pellets only by performing the precision grip, generally using the thumb and the index finger. The board was positioned in front of the monkey with 30° of inclination from horizontal. During each daily session, the monkeys had used both hands. For this task, the analysis is focused on two parameters: i) the number of pellets retrieved by the monkey in the first 30 seconds, counted separately for the vertical slots and the horizontal slots. ii) the contact time (CT), defined as the time (duration) of contact between the fingers and the pellet. It was the time interval between the insertion of the first finger (usually the index finger) into the slot to touch the pellet and the onset of retrieval of the pellet out of the well. The time interval was measured by replaying frame by frame the video sequence. The CT was measured for the first five vertical and horizontal slots aimed by the monkey.

PET-MRI analyses

PET-MRI scanning was conducted in Department of Nuclear Medicine and PET/CT-MRI Center, the First Affiliated Hospital of Jinan University (JNUH) as described in our previous work^37,59,60^. The PET tracer [^18^F] FDG and [^18^F]T807/AV1451 were radiolabeled in the Center of Cyclotron and PET Radiopharmaceuticals (CCPR) in JNUH. Briefly, animals were anesthetized by ketamine (10 mg/kg; i.m.) and placed into the scanner in the supine position for T1-weighted 3-dimensional MR scan. A circular 32-channel array head coil was placed on the top of monkey’s head. Parameters for T1 sequence were as follows: repetition time (TR) = 8.4 ms, echo time (TE) = 3.4 ms, inversion time (TI) = 450 ms, slice thickness = 1.1 mm, matrix size = 256 × 256 and field of view (FOV) = 18 × 18 cm. The volumes of the total brain, prefrontal cortex, hippocampus, entorhinal cortex, caudate and putamen in MRI were calculated by Cavalieri method^61,62^, which is one of the established stereological techniques. Briefly, the converted JPEG image suitable for calculation was automatically placed over the image by the ‘‘Stereo Investigator Software.’’ The areas of brain, prefrontal cortex, hippocampus, entorhinal cortex, caudate, and putamen were selected manually on Stereo Investigator Software. Subsequently, total brain, striatum, and lateral ventricle were calculated by the software program by multiplying the image voxel size with the number of voxels in it.

For PET/CT section, animals were deprived of food for 12–15 h before tracer injection but were allowed to drink water at any time. Each monkey was intravenously injected with ca. 1.0 mL of [^18^F] FDG or [^18^F]T807/AV1451 (18.5 MBq/kg), then kept in a shielded room with minimal ambient noise and light. After 50 min, the subject was immobilized with ketamine (10 mg/kg; i.m.), placed into the scanner in the supine position and maintained under anesthesia with 1–3% isoflurane and 98.4% oxygen. Head position was fixed with a stereotactic frame. CT scan was performed first, followed by eight-minute static positron emission data collection at 60 min post-injection. CT data were acquired in breath-hold with 140 kV, 230 mA modulated using the GE AutomA technique (GE Medical System, Milwaukee, USA) with a noise index of 30, slice thickness of 3.75 mm, slice interval of 3.27 mm, matrix size of 512 × 512 and scan FOV of 50 cm. PET data were acquired in 3-dimensional time-of-flight (TOF) mode with a slice thickness of 3.27 mm, slice interval of 3.75 mm, matrix size of 256 × 256 and scan FOV of 70 cm. The PET data were attenuation-corrected by the integrated CT attenuation-corrected (CTAC). The CT data were reconstructed in standard mode with display field of view (DFOV) of 30 cm and window width/window level of 100/45, advanced statistical iterative reconstruction 40%. The PET data were then reconstructed in terms of the point spread function together with TOF technology with DFOV of 30 cm. Data analysis was performed using PMOD4.1 (Pmod Technologies LLC, Zürich, Switzerland). The co-registration of PET image to individual MR image, which was transformed into brain template MR images, was carried out following published protocols^63^. Volume of interests were extracted from the occipital cortex, temporal cortex, parietal cortex, prefrontal cortex, striatum, thalamus, hippocampus, amygdala, corpus callosum, cingulate, globus pallidus, cerebellum, pons. The uptake of [^18^F] FDG or [^18^F] AV1451 in Pons was used as reference for SUVr analysis as previously described^60^. Statistical analyses were performed with GraphPad Prism 9.0 (GraphPad Software, La Jolla,USA).

Identification of genomic integration sites of transgenes

The Genome-wide distribution of transgene analysis has been well described^64^. In brief, a total of 2 μg genomic DNA was used to construct a DNA library for each case. Sequencing linkers were further added onto genomic segments (length around 500–700 base pairs (bp). After end repairing and 3′ A-adding, the fragmented DNAs were ligated with Y-shape adaptor. Two pair primer: LTR1: 5′-CTTGCCTTGAGTGCTTCAAGTAGT-3′; LTR 2: 5′-TGCCCGTCTGTTGTG TGA CTCT-3′; YP1: 5′-GGATAGCGACGCACGGAACTCT-3′; YP2: 5′-CTCCATCTCATCCTGCG TGTC-3′. Amplification was performed with the adaptor primers. Asymmetry-primer PCR (APP) was used to enrich the viral integration sites in each library. The APP method includes two PCR systems. The first PCR system includes only LTR specific primer. After 12 cycles of linear amplification, adaptor specific primer was added in the PCR system followed by 12 cycles of exponential amplification. PCR products were purified using HiPure Gel Pure DNA Mini Kit (D2111-02, Magen, China). The second PCR system uses a pair of primers nest the primers in the first PCR system. After 12 cycles of linear amplification and 15 cycles of exponential amplification, the PCR products of 500–700 bp in size were isolated by agarose gel electrophoresis before being used to construct libraries with Illumina paired-end adapters according to the manufacturer protocol and sequenced by Illumina MiSeq V3 (2 × 300 base paired ends). Only the paired-end reads showing the fusions of viral sequences and the cynomolgus (Macaca fascicularis) genome segments were selected, in which two mismatches were allowed. The reads showing the same integration position were merged and treated as a unique integration site. Target sequences containing LTR of transgene cassettes and genomic segments flanking the transgenes were analyzed.

Stereotaxic injection

AAV viral injection into monkey brain was performed as reported previously^65^. We injected AAV-GFP and AAV-Tau-P301L into the hippocampus and spinal cord of wild-type monkeys at the age of 5-6 years and 24-25 years (n = 3 each group). Each monkey was anesthetized by intravenous injection of 0.3-0.5 mg of atropine, followed by intramuscular injection of 10-12 mg of ketamine per kg body weight. The monkeys were stabilized on a stereotaxic instrument (RWD, life science, 68901, China), the precise position of hippocampus for stereotaxic injection was determined by MRI before injection. Ten μl of viruses was injected after inserting the needle to certain depth, which was calculated based on the MRI data. For injection of the mouse brain, the C57BL/6 mice at 6 months of age (n = 6 each group, 3 males and 3 females per group) were anesthetized by i.p. injection of 2.5% Avertin, and their heads were placed in a Kopf stereotaxic frame (Model 1900) equipped with a digital manipulator, a UMP3-1 Ultra pump, and a 10 μl Hamilton micro syringe. A 33 G needle was inserted through a 1 mm drill hole on the scalp. Injections occurred at the following stereotaxic coordinates: 3.1 mm posterior to bregma, 1.0 mm lateral to the midline, 4.7 mm ventral to the dura, with bregma set at zero. The microinjections were carried out at the rate of 0.2 μl/min. The microsyringe was left in place for an additional 10 min before and after each injection. The viruses (0.5 μl) of AAV-GFP and AAV-Tau P301L were stereotaxically injected into the right motor cortex of mice. Lumbar puncture was used to injection viruses to the monkey and mouse spinal cord, which was performed at the intervertebral space L3-L4 or L4-L5 using a 23 G needle to inject 50 μl AAV-Tau P301L viruses into the lumen of spinal cord in monkey. For the mouse spinal cord injection, 30G needle was used to inject 3 μl viruses into the lumen.

**References:**

1. Chen, Y., Yu, J., Niu, Y., Qin, D., Liu, H., et al. Modeling Rett Syndrome Using TALEN-Edited MECP2 Mutant Cynomolgus Monkeys. Cell, 169(5), 945-955.e10 (2017).
2. Noser R, Gygax L, Tobler I. Sleep and social status in captive gelada baboons (Theropithecus gelada). Behav Brain Res. Dec 17;147(1-2):9-15 (2003).
3. Rodriguez JS and Paule MG. Working memory delayed response tasks in monkeys. In: Methods of Behavior Analysis in Neuroscience. Second Edition. Buccafusco JJ (ed.). Boca Raton, FL: CRC Press (2009).
4. Darusman, HS., Pandelaki, J., Mulyadi, R., Sajuthi, D., Putri, I. A., et al. Poor memory performance in aged cynomolgus monkeys with hippocampal atrophy, depletion of amyloid beta 1-42 and accumulation of tau proteins in cerebrospinal fluid. In Vivo (Athens, Greece), 28(2), 173–184 (2014).
5. Weed JL, Lane MA, Roth GS, Speer DL, Ingram DK. Activity measures in rhesus monkeys on long-term calorie restriction. Physiol Behav. 62:97–103 (1997).
6. Darusman HS, Sajuthi D, Kalliokoski OH, Jacobsen KR, Call J, et al. Correlations between serum levels of beta amyloid, cerebrospinal levels of tau and phospho tau, and delayed response tasks in young and aged cynomolgus monkeys (Macaca fascicularis). J Med Primatol 42(3): 137-146 (2013).
7. Brinkman, J., and Kuypers, H.G. Cerebral control of contralateral and ipsilateral arm, hand and finger movements in the split-brain rhesus monkey. Brain 96, 653-674, (1973).
8. Schmidlin, E., Kaeser, M., Gindrat, A.D., Savidan, J., Chatagny, et al. Behavioral assessment of manual dexterity in non-human primates. J Vis Exp (2011).
9. Nie B, Wang L, Hu Y, Liang S, Tan Z, et al. A population stereotaxic positron emission tomography brain template for the macaque and its application to ischemic model. Neuroimage. 203:116163 (2019).
10. Xiao Z, Wei H, Xu Y, Haider A, Wei J, et al. Discovery of a highly specific 18F-labeled PET ligand for phosphodiesterase 10A enabled by novel spirocyclic iodonium ylide radiofluorination. Acta Pharm Sin B.12(4):1963-1975 (2022).
11. Ekinci, N., Acer, N., Akkaya, A., Sankur, S., Kabadayi, T., and Sahin, B. Volumetric evaluation of the relations among the cerebrum, cerebellum and brain stem in young subjects: a combination of stereology and magnetic resonance imaging. Surg. Radiol. Anat. 30, 489–494 (2008).
12. Erbagci, H., Keser, M., Kervancioglu, S., and Kizilkan, N. Estimation of the brain stem volume by stereological method on magnetic resonance imaging. Surg. Radiol. Anat. 34, 819–824(2012).
13. McLaren, D.G., Kosmatka, K.J., Oakes, T.R., Kroenke, C.D., Kohama, S.G., et al. A population-average MRI-based atlas collection of the rhesus macaque. Neuroimage, 45, 52–59 (2009).
14. Liu, Z., Li, X., Zhang, J. T., Cai, Y. J., Cheng, et al. Autism-like behaviours and germline transmission in transgenic monkeys overexpressing MeCP2. Nature, 530(7588), 98–102 (2016).
15. Yin, P., Bai, D., Deng, F., Zhang, C., Jia, Q., et al. SQSTM1-mediated clearance of cytoplasmic mutant TARDBP/TDP-43 in the monkey brain. Autophagy, 18(8), 1955–1968 (2022).

Supplementary Figures

Figure. S1.

Figure S1. Generation of Tau-P301L transgenic monkeys, related to Figure 1. (a) Genome-wide distribution of transgenes in Tau monkeys. Insertion sites (dots) distribute on various chromosomes (outermost circle). Sizes of dots are proportional to reads identified by deep-sequencing. (b) Summary of the distribution of transgene insertion sites on chromosome sites.(c) Levels of AD associated biomarkers including aβ40 and aβ42 in plasma and cerebrospinal fluid in Tau transgenic (Tau1, Tau2, Tau3, Tau 5) and wild type (Tau4, 151001, 150064, 160011, and 160077) monkeys at different ages.

Figure. S2.

Figure S2. MRI analysis of Tau monkeys, related to Figure 2. Representative T2-weighted coronal images of live WT (n = 3) and Tau (n = 3) monkeys at approximate 36 months of age. Prefrontal cortex (PFC), hippocampus(Hip), entorhinal Cortex (EC), caudate (Cad), putamen (Put) and lenticular nucleus (Len) are indicated in the images.

Figure. S3.

**Figure S3. Neurodegeneration of Tau1 monkey, related to Figure 4.** (**a**) Anti-AT8 immunostaining of the prefrontal cortex, striatum, hippocampus, and spinal cord of WT and Tau1 monkeys. (**b**) Anti-NFL immunostaining of the cortex, striatum, hippocampus, and spinal cord of WT and Tau1 monkeys. (**c**) Quantification of the numbers of Tau, NFL, and p-Tau positive cells in brain regions in WT and Tau1 (n = 6 fields/each brain section from 6 brain sections). Data are analyzed by Student’s t test and presented as mean ± SEM. *p < 0.05; **p < 0.01; ***p < 0.001.

**Figure S4**

**Figure S4. Neuronal loss in the entorhinal cortex of Tau1 monkey. related to Figure 4.**

(**a**) Western blotting with anti-Flag showing that transgenic Tau is selectively expressed in the monkey brain but not peripheral tissues. (**b**) NeuN staining of the entorhinal cortex of wild type (WT) and Tau1 monkeys.

**Figure S5**

**Figure S6. Axonal degeneration in Tau1 monkey brain, related to Figure 5.** (**a**） Electron microscopy revealing demyelinated axon and reduced myelin layers or defective myelin sheath is evident in Tau1 monkey. (**b**) Western blotting showing the reduced NeuN in the spinal cord of Tau1 and Tau2 monkeys compared with WT monkey.

**Figure S7**

**Figure S7. Toxic Aβ42 in the spinal cord Tau transgenic monkeys, related to Figure 6. (a)** Schematic diagram of the antibody epitope sites in the human APP protein. (**b**) Summary of antibodies used to recognize Aβ42 in Tau monkey brains. (**c**) Dot blotting of the Tau monkeys with Aβ oligomers antibody A11 and 4G8. GAPDH served as a loading control. (**d**) Quantification of the expression ratio of Aβ-oligomer to GAPDH in spinal cord of Tau monkeys. Data are analyzed by One-way ANOVA and presented as mean ± SEM. *p < 0.05; **p < 0.01; ***p < 0.001.

**Figure S8**

**Figure S8. Amyloid-beta staining of the spinal cord of AAV-Tau-injected monkey, related to Figure 7.** Immunofluorescence analysis of the spinal cord of control WT and AAV-Tau-injected monkeys using anti-Flag, anti-Tau, anti-Aβ(6E10), anti-NeuN, and anti-GFAP antibodies.

**Figure S9**

**Figure S9. Selective generation of toxic amyloid-beta oligomers in the spinal cord of monkey, related to Figure 7.** (**a**) Western blotting of the spinal cord of AAV-Tau-injected monkey with multiple anti Aβ1-42 antibodies, D9A3A, ab240360, 6E10, and A11. Red arrows indicate different Abeta42 oligomers. (**b**) Western blotting of the spinal cords of monkeys at different ages, which were injected with the control AAV-GFP or AAV-Tau. The tissue samples were probed with APP C-terminal antibody C1/6.1 and N-terminal antibody sAPPβ. (**c**) Western blotting of the spinal cord of AAV-Tau-injected mouse and monkey using anti-Flag, anti-Tau, and 6E10. Arrow indicates Abeta42 oligomer in the spinal cord of AAV-Tau-injected and Tau1 monkeys, which was not seen the spinal cord of AAV-Tau-injected mice. Vinculin served as a loading control. In (a) and (c), Abeta oligomers are indicated by red arrows.

**Supplemental movies**

Movie 1. Short-term memory test of WT and Tau3 monkeys at 38 months of age.

Movie 2. Long-term memory test of WT and Tau5 monkeys at 40 months of age.

Movie 3. Sleep monitoring of WT and Tau2 monkey at 38 months of age.

Movie 4. Fine finger coordination assay of WT and Tau3 monkey at 30 months of age.

Movie 5. The body movements of Tau1 monkey at 25 and 35 months of age.
